# Retracted: Tanshinone IIA Induces Apoptosis in Human Oral Cancer KB Cells through a Mitochondria-Dependent Pathway

**DOI:** 10.1155/2017/9496485

**Published:** 2017-11-21

**Authors:** BioMed Research International

BioMed Research International has retracted the article titled “Tanshinone IIA Induces Apoptosis in Human Oral Cancer KB Cells through a Mitochondria-Dependent Pathway” [1]. As noted by Amanda Capes-Davis on PubMed Commons, KB cells are cross-contaminated by HeLa and are not oral cancer cells [2]. Therefore, the conclusions cannot be supported. Tan IIA was already known to induce apoptosis in HeLa cells through a mitochondria-dependent pathway [3].
